# Hotspots and main drivers of fecal pollution in Neusiedler See, a large shallow lake in Central Europe

**DOI:** 10.1007/s11356-018-2783-7

**Published:** 2018-08-13

**Authors:** István G. Hatvani, Alexander K. T. Kirschner, Andreas H. Farnleitner, Péter Tanos, Alois Herzig

**Affiliations:** 1 Institute for Geological and Geochemical Research, Research Centre for Astronomy and Earth Sciences, Hungarian Academy of Sciences (MTA), Budaörsi út 45, Budapest, H-1112 Hungary; 2 Institute for Hygiene and Applied Immunology—Water Hygiene, Medical University Vienna, Kinderspitalgasse 15, A-1090 Vienna, Austria; 3 Karl Landsteiner University for Health Sciences, Dr.-Karl-Dorrek-Straße 30, A-3500 Krems, Austria; 4 Interuniversity Cooperation Centre Water & Health, Vienna, Austria; 5 Technische Universität Wien, Research Centre for Water and Health 057-08, Institute for Chemical, Environmental and Bioscience Engineering, Gumpendorferstrasse 1a, A-1060 Vienna, Austria; 6 Department of Mathematics and Informatics, Szent István University, Páter Károly utca 1, Gödöllő, H-2100 Hungary; 7 Biological Research Institute Burgenland, A-7142 Illmitz, Austria; 8 Nationalpark Neusiedler See-Seewinkel, A-7143 Apetlon, Austria

**Keywords:** Large lake, Microbial fecal pollution, Monitoring, Principal component analysis, Stochastic modeling, Variography, Kriging

## Abstract

To minimize the risk of negative consequences for public health from fecal pollution in lakes, the continuous surveillance of microbiological water quality parameters, alongside other environmental variables, is necessary at defined bathing sites. Such routine surveillance may prove insufficient to elucidate the main drivers of fecal pollution in a complex lake/watershed ecosystem, and it may be that more comprehensive monitoring activities are required. In this study, the aims were to identify the hotspots and main driving factors of fecal pollution in a large shallow Central European lake, the Neusiedler See, and to determine to what degree its current monitoring network can be considered representative spatially. A stochastic and geostatistical analysis of a huge data set of water quality data (~ 164,000 data points, representing a 22-year time-series) of standard fecal indicator bacteria (SFIB), water quality and meteorological variables sampled at 26 sampling sites was conducted. It revealed that the hotspots of fecal pollution are exclusively related to sites with elevated anthropogenic activity. Background pollution from wildlife or diffuse agricultural run-off at more remote sites was comparatively low. The analysis also showed that variability in the incidence of SFIB was driven mainly by meteorological phenomena, above all, temperature, number of sunny hours, and wind (direction and speed). Due to antagonistic effects and temporal undersampling, the influence of precipitation on SFIB variance could not be clearly determined. Geostatistical analysis did reveal that the current spatial sampling density is insufficient to cover SFIB variance over the whole lake, and that the sites are therefore in the most part representative of local phenomena. Suggestions for the future monitoring and managing of fecal pollution are offered. The applied statistical approach may also serve as a model for the study of other such areas, and in general indicate a method for dealing with similarly large and spatiotemporally heterogeneous datasets.

**Electronic supplementary material:**

The online version of this article (10.1007/s11356-018-2783-7) contains supplementary material, which is available to authorized users.

## Introduction

Lakes in the temperate climate zone are intensively used for recreational purposes. The changes witnessed in the ecological status of European lakes over the second half of the twentieth century severely impacted their usability for water supply, recreation, and tourism (Dokulil 2014; MEA 2005) and their ecosystem services. However, due to the comprehensive measures, e.g., (1) the construction of sewage systems, waste water treatment plants (WWTPs) (see, for example, Hatvani et al. 2014a), (2) the introduction of natural protection zones, e.g., Hatvani et al. (2014b), and (3) the implementation of the EU Water Framework Directive (EU-WFD) (EC 2000) etc., water quality has improved to a marked degree in recent decades. With regard to fecal pollution, specific water quality targets have been implemented in legislation which demands the surveillance of water quality on a regular basis to enable safe recreational water use (EU Bathing Water Directive, EU-BWD, EC 2006). In addition to surveillance, a fundamental understanding of potential sources of pollution and of the factors influencing pollution patterns in lakes is a prerequisite for effective water management. In all European Union countries, the surveillance of microbiological water quality extends over the summer bathing season and encompasses the enumeration of standard fecal indicator bacteria (SFIB) *Escherichia coli* and intestinal enterococci, based on international standard procedures; this is conducted on a relatively small number of occasions at defined time intervals at nominated bathing water sites (EC 2006).

The main aim of the directive has been to force EU member states to minimize the risk of negative consequences for public health from fecal pollution in bathing waters through improved water monitoring and management practices (Bedri et al. 2016). Due to (1) the increased awareness of water quality deterioration in lakes and rivers (Strobl and Robillard 2008), and (2) developments in analytical and monitoring equipment, the number of monitoring sites and water quality parameters assessed (physical, chemical, and biological) has increased in rivers (Chapman et al. 2016) and lakes (Kovács et al. 2012b) as well. This has, in turn, led to increased opportunities to spot and manage fecal pollution in waters used for recreation. However, improper monitoring practices and the under-exploitation of the information contained in the data clearly undermine these aims (Ward et al. 1986). The proper planning of monitoring strategies for rivers and lakes—e.g., Bartram et al. (1996), Chapman et al. (2016), Strobl and Robillard (2008)—and the thorough statistical processing of water quality data from activities related to the EU-WFD and EU BWD is essential if representative information is to be obtained (Hatvani et al. 2014b; Wymer 2007). This is a vital requirement in international guidelines for bathing water management (WHO 2003.)

In Austria, since the effective implementation of wastewater management in the 1970s and 1980s, the chemical and microbiological water quality of lakes has significantly improved (Dokulil 2017). Besides the construction of WWTPs, water management is based on the rerouting of contaminated wastewater away from the lake ecosystem and the discharge of the treated wastewater into flowing waters (rivers). The situation at the Neusiedler See, the western-most brackish lake in Europe and the largest lake in Austria, is, however, different (Magyar et al. 2013). The lake is under the de-jure protection of the Ramsar Convention (Convention on Wetlands 1971) and lies within the territory of national parks in both Austria and Hungary; furthermore, the site gained World Heritage status in 2001 (Dinka et al. 2016). Nonetheless, it receives treated wastewater directly from four WWTPs (one on the Hungarian part of the lake). The outflow of further three WWTPs enters the lake through the waters of the River Wulka (Magyar et al. 2013). Moreover, unrecognized point and non-point sources (agricultural run-off, livestock, and wildlife) may also add to the fecal pollution of the lake. According to the EU-BWD (EC 2006), routine surveillance of bathing water sites in the lake is restricted annually to five samples, the minimum required, collected during the bathing season for four consecutive years to obtain a classification of the bathing water quality. Prior to the implementation of the revised EU-BWD directive into Austrian national legislation (Badegewässerverordnung 2009, BGBl II/349), different microbiological parameters such as fecal coliforms, total coliforms, or streptococci were used by different member countries, while surveillance practices differed considerably between them.

Since routine surveillance does not necessarily allow an understanding of the observed complex patterns of fecal pollution in the lake, a much more detailed monitoring of microbial water quality parameters and their relation to potential influencing factors (e.g., meteorological parameters) is required. Thus, in order to expand the routine surveillance of bathing sites during the bathing season at Neusiedler See, an extended set of parameters for microbiological water quality (SFIB) covering 26 critical sampling sites around the lake over a period of 22 years was examined. Samples were taken not only during the bathing season (June 1st to August 31st) but during the period from March 1st to October 31st, when several recreational activities take place (surfing, sailing, fishing, etc.), in the course of which visitors come in direct contact with the water. Preliminary observations did shed light on a few alarming water quality events and a large data set has been collected over time. Still, no thorough statistical investigation has been conducted to assess the lake-wide patterns and driving factors of SFIB variance in order to guide future monitoring and management practices.

Therefore, stochastic and geostatistical analyses were employed (1) to identify the hotspots of fecal pollution in the Neusiedler See based on SFIB abundance, (2) to elucidate the main driving factors of the fecal pollution, and (2) to assist in the optimization of the current spatiotemporal state of the monitoring system with respect to its spatial sampling frequency. With this comprehensive approach, a better management of the microbiological water quality of Neusiedler See should be possible, and it is hoped that this study will serve as a model for comparable lake ecosystems.

## Materials and methods

### Study area description

The Neusiedler See (47°42′ N, 16°46′ E) is the largest (full area 309 km^2^) shallow (average water depth 1.1 m) lake in Austria, though a quarter of this area actually lies within the territory of Hungary (75 km^2^) (Dinka et al. 2016). Its main water inputs are precipitation (average 181 × 10^6^ m^3^ year^−1^, 78% for 1967–2012; Kubu et al. 2014) and the two small natural streams, the Wulka (Austria, average discharge 42 × 10^6^ m^3^ year^−1^, 14%) and the Rákos (Hungary; average discharge 2.5 × 10^6^ m^3^ year^−1^, 1%); the Golser Canal (Austria, average discharge 7 × 10^6^ m^3^ year^−1^, 2.5%) also plays a role. Its output is mainly due to evaporation and evapotranspiration (208 × 10^6^ m^3^ year^−1^, 90%). About 10% of its shoreline is affected by anthropogenic activity. In periods of high water levels (above 115.8 m.a.s.l.), a channel is activated that allows the run-off of surplus water (26 × 10^6^ m^3^ year^−1^, 10%). There is almost constant air movement in the area, and thanks to orographic effects the prevailing wind direction is NW (Józsa et al. 2008). The lake has dried out many times over the centuries. Therefore, water management measures are continuously applied, with a considerable influence on the current form of the reed stands which cover about 56% of the lake (Magyar et al. 2013).

A major driver of the microbiological-hygienic status of the lake is the inflow of treated wastewater (Kirschner et al. 2014). Several indirect and direct wastewater influents from WWTPs into the lake exist around the lake (Table S1). The major indirect influent is the Wulka, with approximately 220,000 population equivalents (PE). The major direct influent is the WWTP at Podersdorf (PE 20,000), which is the only WWTP equipped with UV disinfection. All of these influents pass through the reed stands. However, the width of the reed stands on the west side of the lake is much greater than on the east side. In the west, they extend over 2–3 km, receiving the Wulka, the effluents of Jois (PE 8400) and Balf (PE 4000), while in the east they extend only over 50–100 m, receiving the Golser Canal (PE 40,000) and the effluents of Podersdorf. In the case of all WWTP influents, sewer overflows in conditions of heavy rainfall are considered as potential main pollution events for the lake (Sommer et al. 2018). In addition to the WWTPs, the inflow of polluted water may derive from smaller channels draining agricultural areas (mainly cattle and horse raising), from surface run-off during rainfall events, and from wildlife (mainly birds).

### Description of sampling sites

**Fig. 1:**
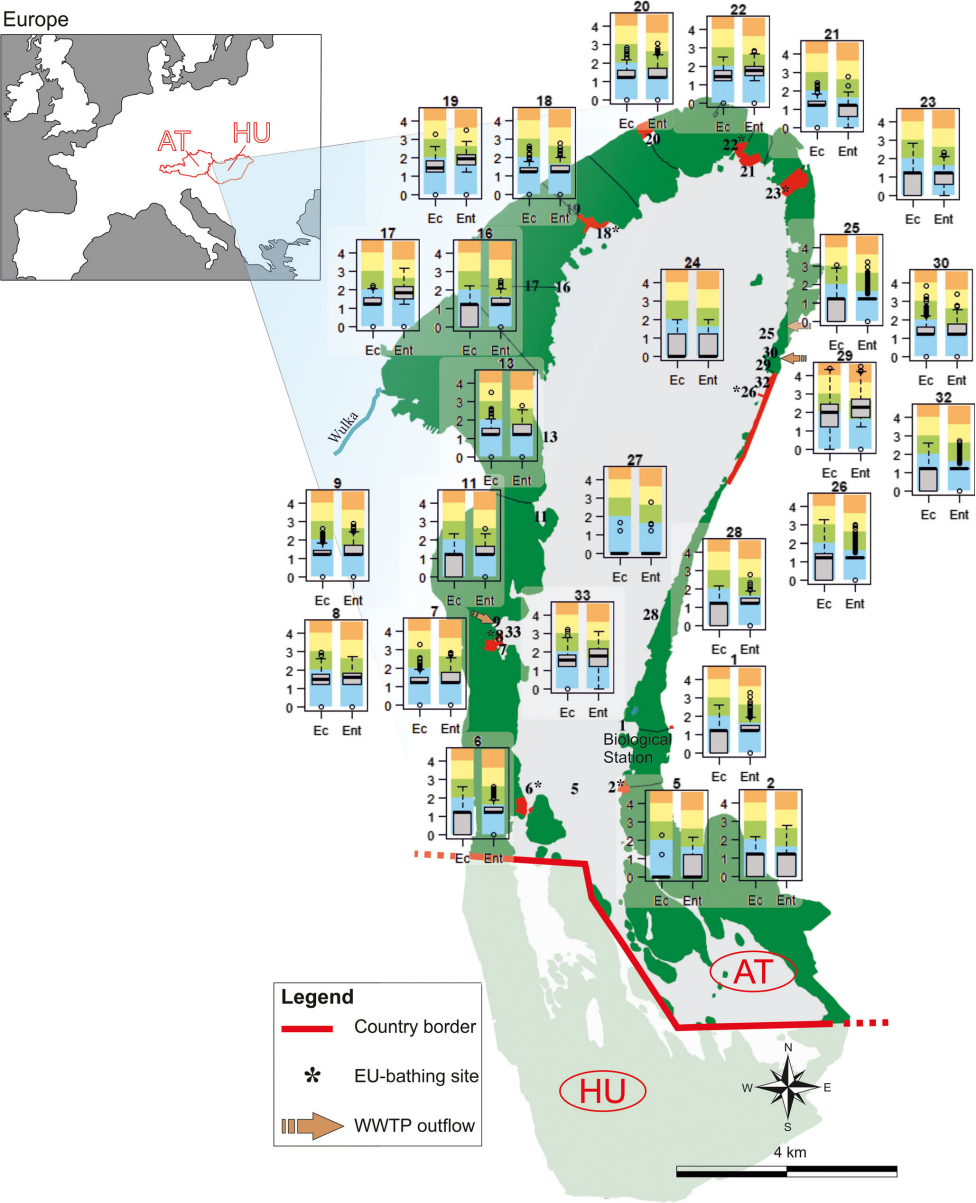
Boxplots of EC and ENT concentrations at the 26 monitoring sites of Neusiedler See (1992–2013) on a logarithmic scale. Classes of fecal pollution are shown as blue (little), green (moderate), yellow (critical), and orange backgrounds (strong pollution), after Kirschner et al. (2009, 2017). Data falling outside a value of 1.5 times the interquartile range are indicated with a circle (outliers and extreme values); the black lines in the boxes represent the median

In total, eight official EU bathing sites are situated around the Neusiedler See, seven in Austria, in the municipalities of Mörbisch, Rust, Breitenbrunn, Neusiedl, Weiden, Podersdorf and Illmitz, and one in Hungary, Tavi-Vizitelep (EEA 2018). In this study, only the Austrian sites were considered because for the Hungarian part of the lake, the set of parameters assessed, the sampling strategy, and the analytical methods used differed from the Austrian ones, rendering their joint handling impossible. In addition to the seven Austrian official bathing sites, further 19 sites were selected for the monitoring of microbial fecal pollution, those having sufficient available data for the whole investigation period, 1992–2013 (Table S1; Fig. 1). They included potentially critical sites of microbial fecal pollution and a few reference sites with “background” levels of fecal pollution.

### Acquired dataset and preprocessing

#### Water quality data

The time series of the analyzed water quality parameters, including SFIB data, was acquired from the Biological Research Institute Burgenland at Illmitz. The presence of intestinal enterococci was determined in accordance with ISO 7899–2:2000. From 2003 onwards, *E. coli* concentrations were determined using the Colilert-18 system (IDEXX, Ludwigsburg, Germany), which became an ISO standard procedure in 2012 (ISO 9308–2:2012). Prior to 2003, in the place of *E. coli*, thermotolerant coliforms were recorded, in accordance with EN ISO 9308–1:1990 (Part 1, membrane filtration method). In 2003, both methods were run in tandem. Due to this change in the analytical method, a conversion to Colilert based on the parallel measurements of data dating from before 2003 had to be made; for details see Electronic Supplementary Material (ESM) Section “Conversion of *E.coli* concentration.” All SFIB are expressed in CFU (colony forming units, Enterococci) or MPN (most probable number, *E.coli*) per 100 ml and log_10_ transformed for statistical analysis. The other water quality parameters examined were water temperature (*W*_Temp_; °C), pH, electric conductivity (Cond.; mS cm^−1^), dissolved oxygen (DO), total phosphorus (TP), ammonium-N, nitrate-N, chlorophyll-*a* (all mg l^−1^), and Secchi depth (cm), all collected at 26 sampling sites (Fig. 1). During winter, no sampling was conducted at many monitoring sites. Thus, for each year from 1992 to 2013, all the data (water quality and SFIB) measured between March 1st and October 31st (at weekly/bi-weekly/monthly intervals, depending on the station and the year) were averaged for each year and included in the analysis. The same procedure was conducted on the water level data (cm above Adriatic Sea level) recorded daily at six sites across the lake (Apetlon, Breitenbrunn, Illmitz, Mörbisch, Podersdorf, and Rust), along with the daily discharge data (*Q*; m s^−1^) of the Wulka, obtained from the Hydrographical Services of the Province of Burgenland. The whole SFIB and water quality dataset consists of ~ 8700 observations, including ~ 14,700 SFIB and 93,600 water quality measurements (108,300 measurements in total). It should be noted that instead of annual averages, the median or even percentiles could have been used in the analyses. However, in the case of percentiles, the outlying and extreme values would have biased the results, providing a not representative picture of the actual variability of water quality in the lake. In the case of using the medians, a similar pattern would have been obtained as with the used averages since they were almost identical at most of the sites for most of the parameters (e.g. Fig. S1).

#### Meteorological data

The time series of the meteorological variables (air temperature (°C), precipitation (mm day^−1^; 7:00 – 7:00 next day), wind speed (m s^−1^ daily avg.), wind direction, hours of sunshine, global radiation (J cm^−2^)) were retrieved from six meteorological stations (Table S2) for the years 1992–2013. The original data were modified only in the case of wind direction to facilitate further data analysis; for details, please see ESM Section “Wind direction conversion.” The data sets were obtained from the Austrian Centre for Meteorology and Geodynamics (Zentralanstalt für Meteorologie und Geodynamik) and the Biological Research Institute of Burgenland. The whole meteorological dataset consists of ~ 56,000 measurements.

### Methodology and statistical approach

The complexity of the research question, the identification of the driving factors of SFIB variance in a shallow lake, and the determination of the spatiotemporal heterogeneity of the dataset called for a novel methodological approach. For this purpose, the ~ 164,000 individual data points (section “Acquired dataset and preprocessing”) were organized into matrices. In this way, principal component analysis could be employed on the SFIB records of all the monitoring sites to identify any hotspots of SFIB variance. Thus, it was also consequently possible to examine the relationship of the obtained principal components—which accounted for most of the SFIB variance in the lake—to the environmental parameters. In addition, via the exploitation of the spatial characteristics of the SFIB data, the spatial representativity of the monitoring network could be assessed through the use of variography (Chilès and Delfiner 2012). The methodology presented serves as an example for other study areas with regard to the question of how similarly large and spatiotemporally heterogeneous datasets should be handled.

#### Principal component analysis

Principal component analysis (PCA) was used to find the sampling sites with the greatest degree of influence on microbial fecal pollution in the lake over the investigated time period. PCA decomposes the original dependent variables into principal components that explain the original total variance of the dataset in a component-wise monotonically decreasing order. The correlation coefficients between the original parameters and the principal components (PCs) are the factor loadings. They explain the weights of the PCs in the original parameters (Tabachnick and Fidell 2014).

In the present case, the input variables for PCA were the annual averages (1992–2013) for *E. coli* (EC) and intestinal enterococci (ENT) at each sampling site to make the available data comparable due to the highly variable frequency of sampling and the gaps in it. Thus, the obtained factor loadings of the PCs provide information on the importance of each site regarding fecal pollution in the lake over the whole investigated time interval. The sites with factor loadings above 0.7 delineate those areas responsible for most of the microbial fecal pollution variance, and these will be designated “hotspots”. The PCs were taken into account based on their scree plots (Cattell 1966) and their eigenvalues, which had to be above 1 (Kaiser 1960).

**Fig. 2:**
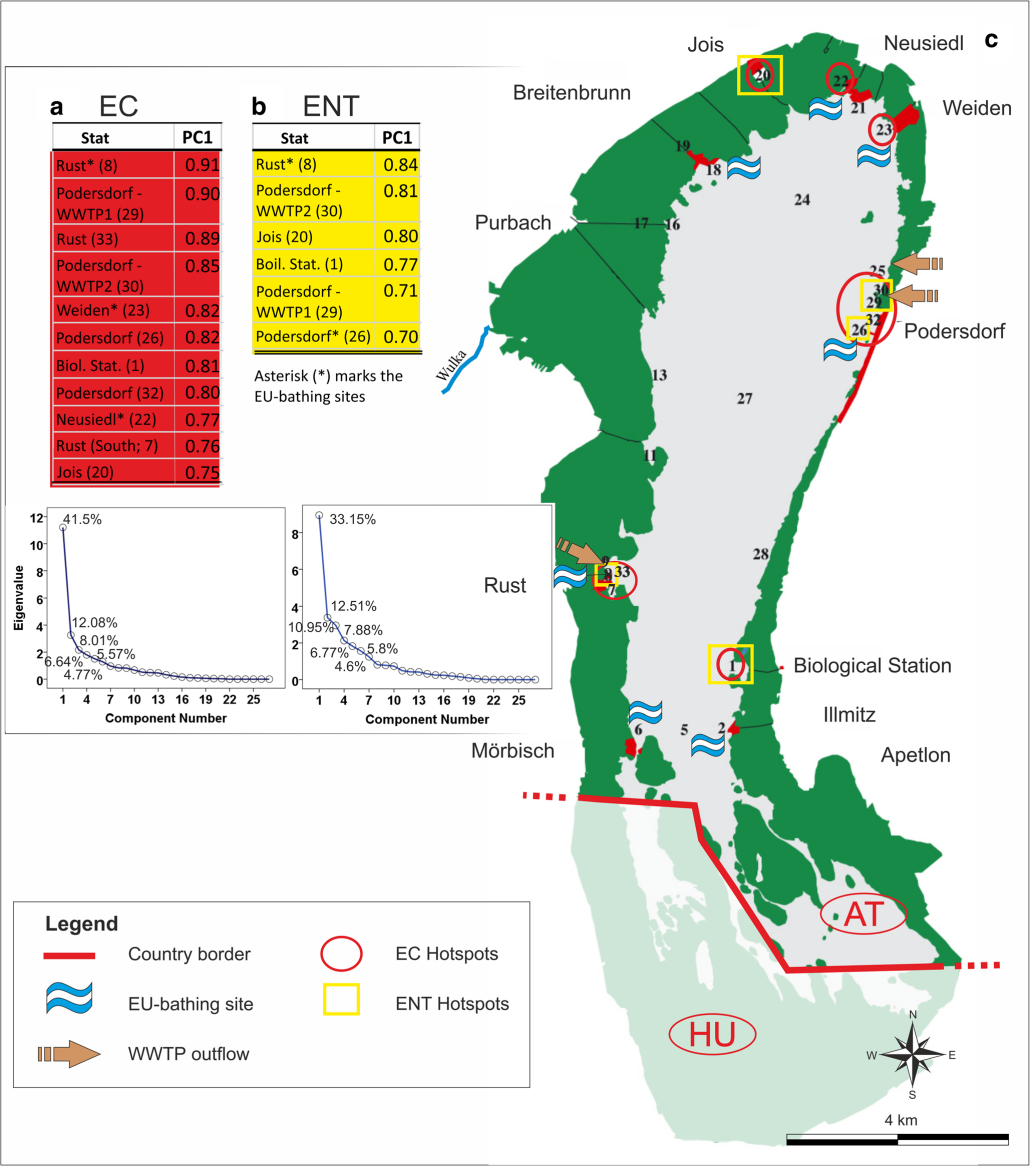
The loadings and the scree plot of the first PC at the different sampling sites for EC (**a**) and ENT (**b**), and the overall pattern of fecal pollution in the Neusiedler See with the EC (red circle) and ENT (yellow square) hotspots, along with all the sampling sites (**c**). Loadings above the chosen 0.7 threshold are marked in red and yellow for EC (**a**) and ENT (**b**), respectively. The numbers on the map (**a**) and in the brackets (**b**, **c**) represent the sampling sites. The scree plots can be found below tables (**a**) and (**b**) for EC and ENT respectively, with the explained variance of the PCs having an eigenvalue > 1

Subsequently, it was explored whether a relationship exists between the various water quality, hydrographical and meteorological parameters taken as independent variables, and the PCs of EC and ENT as dependent ones. For example, the time series (scores) of the first PCs for EC and ENT were correlated separately with the annual averages of the independent variables measured at those sampling sites which were hotspots of a particular SFIB (Fig. 2). If a significant linear relationship is found to exist, a particular independent variable may then be considered to be a driving factor of microbial fecal pollution over the period 1992–2013; in such a case, the greatest impact would be at the hotspots.

#### Variogram analysis

Spatial sampling frequency analysis was conducted on the SFIB data for the whole time period investigated with the aim of (1) objectively determining whether the current sampling grid is sufficient or not, and if the grid was insufficient, (2) offering suggestions concerning the recalibration of the network. For this purpose, a geostatistical variogram analysis (Chilès and Delfiner 2012) was carried out, following Hatvani et al. (2017) and Kovács et al. (2012a). The semivariogram describes the spatiotemporal correlation structure of the data. When the distance *h* between the samples grows (in time or space), the semivariogram values are expected to increase. This implies that the expected difference between the measured responses of the point pairs will also increase (Eq. 1 and Fig. S6).

The core of the semivariogram’s delineation is as follows:

Let *Z*(*x*) and *Z*(*x* + *h*) be the values of a parameter sampled at points at a distance |*h|* from each other. The value of *h* may be measured in time or space for temporal or spatial processes, as required. The semivariogram may then be calculated using the Matheron algorithm (Matheron 1965) (Eq. 1):

1 $$ \gamma (h)=\frac{1}{2N(h)}{\sum}_{i=1}^{N(h)}{\left[Z\left({x}_i+h\right)-Z\left({x}_i\right)\right]}^2 $$

where *Z*(*x*) and *Z*(*x* + *h*) are the values of a parameter sampled at a distance |*h|* from each other, and *N*(*h*) is the number of pairs within the lag interval *h.*

The most important properties of the semivariogram are (1) the “nugget” *C*_0_, (2) the sill, which is the level at which the semivariogram stabilizes (*C* = reduced sill + *C*_0_), and (3) the range (*a*), the distance within which the samples still have an influence on each other as depicted in Fig. S6 (Webster and Oliver 2008). If the semivariogram does not have a rising section, the empirical semivariogram’s points will align above the abscissa and parallel to it. A semivariogram like this is called a nugget-effect type of semivariogram, one for which no range can be estimated, i.e., the sampling frequency is insufficient (Hatvani et al. 2017). Samples outside the semivariogram range (be it temporal or spatial) are considered as uncorrelated (Chilès and Delfiner 2012). Thus, the interdependence structure remains undetectable. Therefore, in order to be able to describe the processes, samples should be taken inside the spatial, or—as in this case—temporal range.

In terms of the practical technique, the empirical semivariograms were first derived, then the best-fit theoretical ones modeled in GS+ geostatistical software, as forcefully suggested by Oliver and Webster (2014) and as put into practice by Hatvani et al. (2017). These best-fit model semivariograms were consequently used to provide the weights in the interpolation of the contour maps using Kriging (Chilès and Delfiner 2012; Cressie 1990).

### Software used

All statistical computations were performed using the authors’ own scripts written in R 3.2.3 (R Core Team 2018), and Visual Basic for MS Excel 2016, while the geostatistical computations and mapping were carried out with Golden Software Surfer 15 and GS+ 10. For the visualizations of the results, CorelDRAW Graphics Suite X8 and MS Office 2016 were used.

## Results

### Overall pattern of the variance of the fecal indicator bacteria in the lake

An empirical inspection of the SFIB concentrations at the different sampling sites around the lake revealed substantial differences between the sites (Fig. 1). Open water sites (e.g., sites 5, 24, 27) showed little fecal pollution while occasionally critical to strong pollution levels were observed at specific stations next to settlements (e.g., sites 7, 33, 29, 30). At the open water sites, the averages of EC and ENT values were at their lowest, while the coefficients of variation (CV) were highest. The mean and the median values indicate that this is only due to occasional pollution events, a fact also reflected in the high statistical ranges (Table 1). At the highly polluted sites (e.g., site 29), low CV and high average values were observed, indicating a quasi-continuous SFIB load, while at site 11 both the average and the CV of the SFIB values are low, indicating continuous little to moderate fecal pollution (Table 1).

**Table 1.**
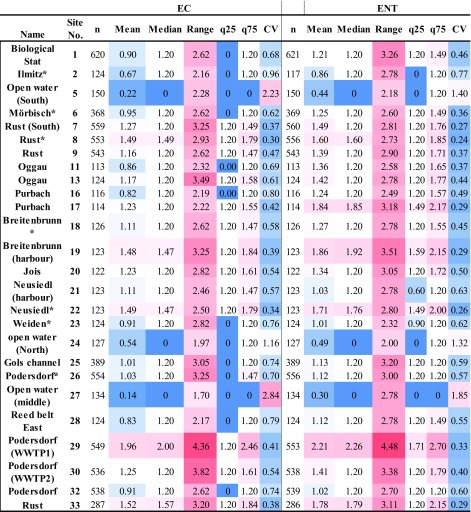
Descriptive statistics of annual averages of EC and ENT values in Neusiedler See (log_10_-transformed data). SD stands for standard deviation and CV for coefficient of variation. At all sites and in all cases the minimum value was zero, thus the range determines the maximum values as well. The darker red shades indicate higher, while the darker blue shades lower values. Asterisks (*) mark the EU bathing sites; for more information on the sampling sites, see Tables S1 and S3 and Fig. S1

The 75th quantiles were far from the maximum values at sites near settlements, especially at site 29, where the maximum is nearly twice as high as the 75th quantile, indicating occasional extreme fecal inputs. The average pollution levels at the monitoring locations around site 29 (sites 26, 32) are much lower than at site 29, implying that the fecal pollution from the WWTP effluent is mainly restricted to a small area. At site 26, a relatively high statistical range was observed, most probably due to the close vicinity (< 1 km) of the WWTP effluent (site 29).

In general, the seven EU bathing sites had good water quality with little fecal pollution levels, as reflected in low mean and median values (Table 1, Fig. 1). For a comparison of the data with the guideline values of the EU-BWD, the 90th and 95th percentiles were calculated for all data (period March till October) and for the bathing season only (June–August) using individual observations (Table S3). These data corroborated the good water quality at the seven EU bathing sites. At all bathing sites, the guideline values for good bathing water quality were met, and five of these sites (sites 2, 6, 18, 23, and 26) even complied with the guideline values for excellent water quality. No difference was observed between the two time periods considered (Table S3). Out of the other sampling sites, only four (sites 17, 19, 29, and 33) exceeded the limit values of good bathing water quality, and most of the sites (13 out of 19) complied with the guideline values (EC 2006) for excellent water quality (Table S3).

### Main driving factors of fecal pollution in the Neusiedler See

#### Fecal pollution hotspots in the lake determined using principal component analysis

The first PCs of EC and ENT explained ∼42% (Fig. 2a) and ∼33% (Fig. 2b) of the SFIB variance in the lake (Fig. 2c), respectively. In all further interpretations, only the first PC was considered in this study, as indicated clearly by the scree plots (Fig. 2a,b). With PCA, the hotspots (PC loadings > 0.7) were determined in the lake for both EC and ENT (Fig. 2a, b). It thus became clear that these hotspots account for most of the EC and ENT variability in the lake. It should be noted here that more sites are considered as hotspots in relation to EC than to ENT, but all the sites which were ENT hotspots (Fig. 2b) were also EC hotspots (Fig. 2c). All of these hotspots are without exception located near settlements; none can be found in the reed channels or in the open water (Fig. 2c). The hotspot with the most sites involved was found at Podersdorf (site 29), where all four investigated sites were EC and ENT hotspots, with the exception of site 32 for ENT.

#### Relationship between the EC and ENT hotspots and the independent variables

To investigate which independent variables might be driving the EC and ENT variance at the hotspots (Fig. 2c), the scores of the first PCs of EC and ENT were correlated with the annual averages of the independent variables’ time series, in a way similar to that applied in Hatvani et al. (2015) and Kovács et al. (2015), in both cases using dynamic factor analysis. As far as all the independent water quality and the meteorological parameters go, it was found that water temperature, precipitation at Rust, air temperature, and the number of sunny hours showed a significant negative correlation with the first PC of EC. In the case of ENT, water temperature and precipitation at Rust were negative correlates (Table 2). A positive linear relationship between SFIB and the independent variables was only observed between average- (vv) and maximum wind speed (vv_max_) recorded at Neusiedl and nitrate-N (Table 2). In the case of ENT, the correlation coefficients did not even approximate *r* = ± 0.7. The water levels and run-off measured at the Wulka, along with global radiation and other parameters not listed in Table 2, were insignificantly related to the PCs.

**Table 2:** Significant correlation coefficients between the independent variables’ time series and the EC and ENT PC1 scores

	EC	ENT
*W*_T_	− 0.43**	− 0.46**
NO_3_-N	0.65**	0.41*
*T*_air_	− 0.43**	–
Prec_Rust_	− 0.47**	− 0.56**
Sunny hours	− 0.50**	–
vv	0.69**	0.41*
vv_max_	0.4*	–

Significance levels: *α* = 0.05 (**); *α* = 0.1 (*)

Following the observation that the PCs of the SFIB showed a high and significant degree of correlation with the average and maximum wind speeds, it was investigated to what degree wind speeds from different points of the compass (directional wind speeds) correlate with the PCs of EC and ENT taken separately. By correlating the first PCs for EC and ENT with these directional wind speeds (derived as described in ESM Section “Wind direction conversion”), it was found that with the exception of South, the other directions all have a significant linear relationship with both SFIBs (Table 3).

**Table 3:** Correlation coefficients between the scores of the first PCs for EC and ENT for the hotspots and the directional wind speed time series attributable to the various wind directions

	EC	ENT
N	0.69**	0.39*
S	0.07	0.11
E	0.68**	0.54**
W	0.82**	0.53**

Significance levels: *α* = 0.05 (**); *α* = 0.1 (*)

**Fig. 3:**
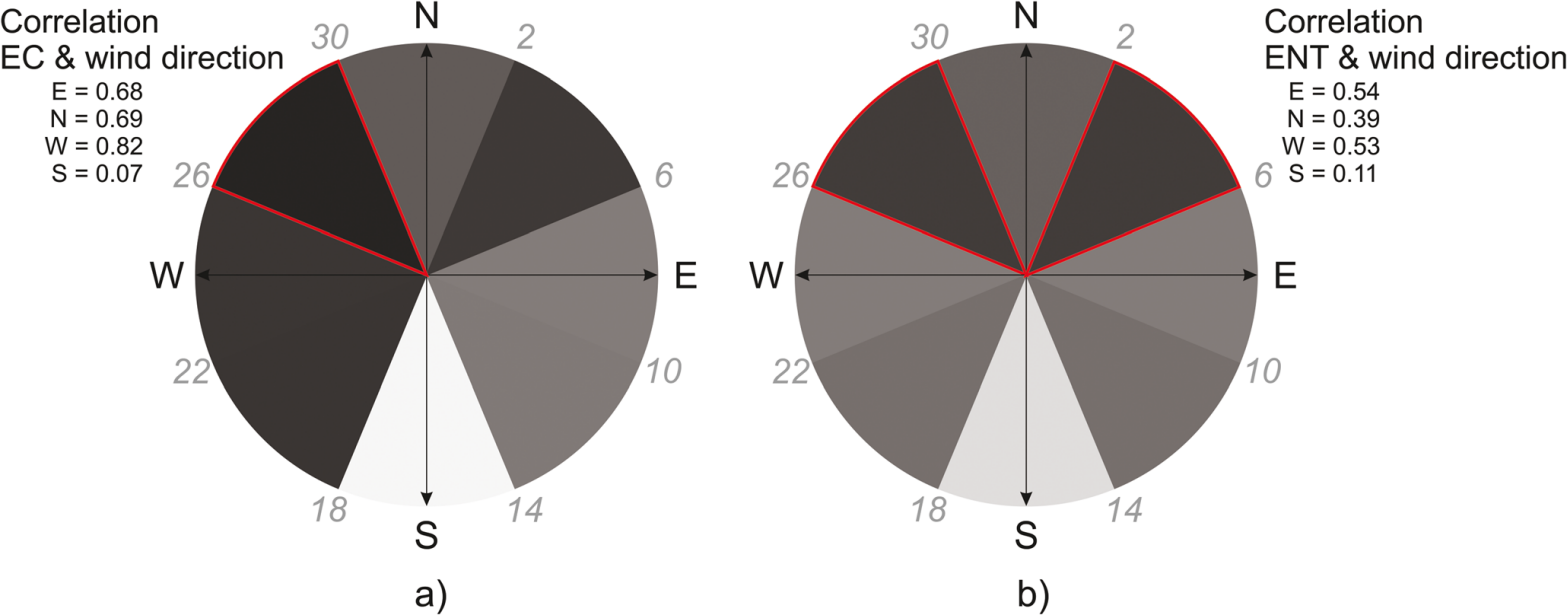
Overlaid correlations between the annual wind speeds attributed to various wind directions (for details see ESM Section “Wind direction conversion”) for EC (**a**) and ENT (**b**). Red triangles mark the wind directions which determine to the greatest degree. The color of the graph is scaled to the magnitude of the correlation coefficient (e.g., *r* = 0.82 corresponds to 82% black). N, North; E, East, W, West; S, South

Plotting the correlation coefficients of the first PCs of the SFIB and the directional wind speeds (Fig. S5), the most determining directional wind speeds were identified. In the case of EC, the North-West was the most dominant (Fig. 3a), while in the case of ENT, both the North-East and North-West were important, and this can be seen to be related to the SFIB variance in the lake (Fig. 3b), suggesting the prevailing importance of Northerly winds (see ESM Section “Wind direction conversion” for details).

### Spatial sampling frequency estimation

**Fig. 4:**
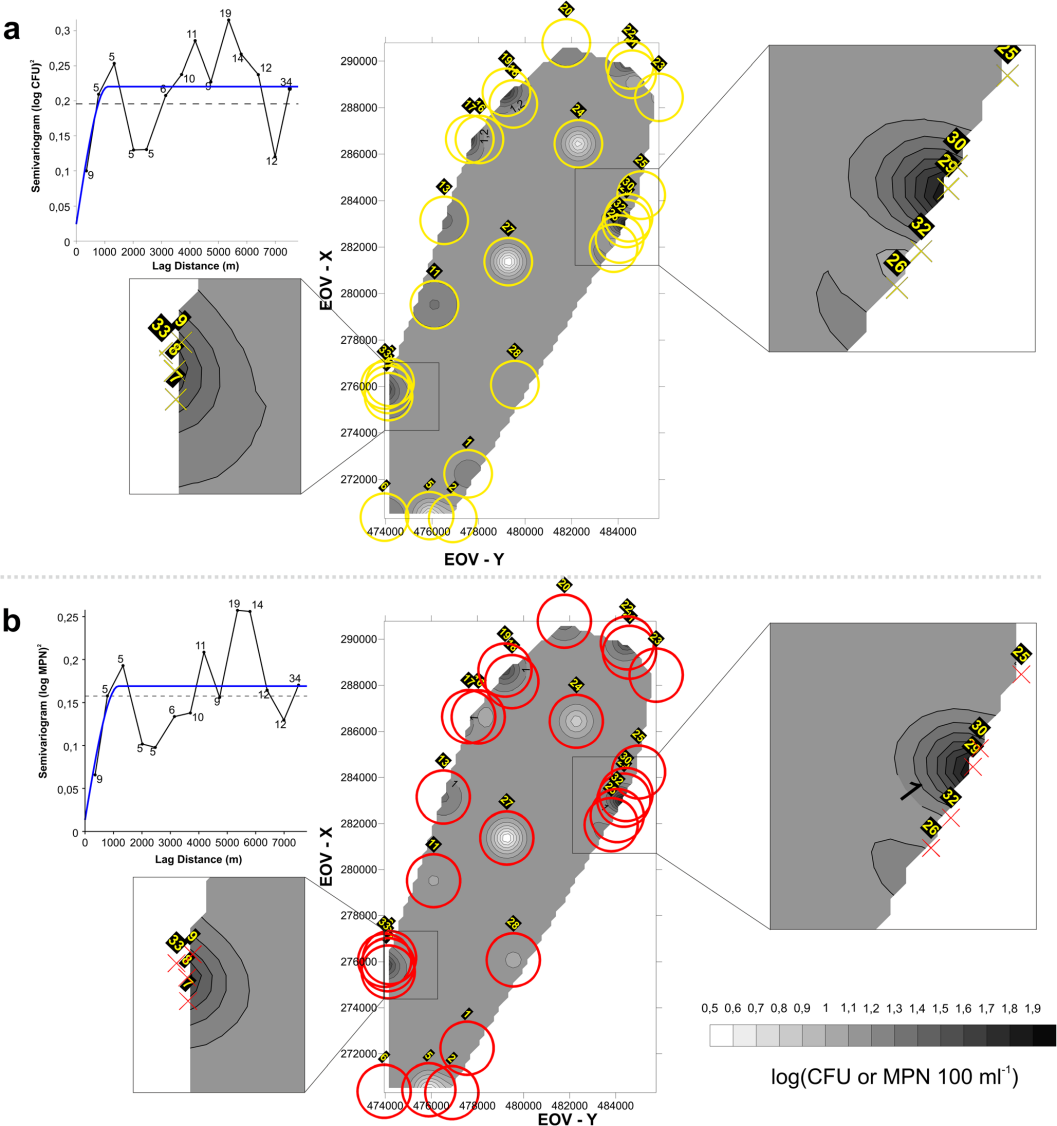
Empirical (black) and theoretical (red) semivariograms (left) and the kriged isoline maps (right) for **a** ENT and **b** EC. The parameters of the isotropic semivariogram models for ENT were *C*_0_ = 0.0242; *C*_0_ + *C* = 0.2204; *a* = 1110 m and for EC: *C*_0_ = 0.0135; *C*_0_ + *C* = 0.169; *a* = 1180 m. The semivariograms and the maps represent the 1992–2013 interval and the sites chosen for this study. Colored circles represent the isotropic sampling ranges around the sites for ENT (**a**; yellow) and EC (**b**; red) in accordance with *a* (the range; see “Variogram analysis” section). The horizontal broken line on the semivariogram plots marks the variance, and the numbers indicate the number of data pairs that was used to derive the semivariogram value for a particular bin. For further theoretical information, see Fig. S6. The maps were produced using the Hungarian National Projection System (EOV; NLCO ( 1971))

With the application of variography, it was possible to see that the current sampling grid of the sites is too underdeveloped to obtain a spatially continuous geostatistical picture of the whole lake. For ENT, a 1.1 km (Fig. 4a), while for EC a 1.2 km spatial range (Fig. 4b) was modeled. For the two parameters, the kriged maps show a quite similar pattern, dominated by so-called bull’s eyes. These indicate that the sites are representative of mostly local phenomena, and further suggesting that interpolation between the sites was insufficient (Chilès and Delfiner 2012). Isolines were plotted only at the multisite hotspots (e.g., Rust, Podersdorf), where the density of the sampling sites was sufficient.

## Discussion

The regular input of fecal pollution from WWTPs or receiving waters may continuously impair the water quality of lakes, e.g., the Great Lakes (Nevers et al. 2014) or Lake Geneva in Switzerland (Poté et al. 2009). Short-term fecal pollution events driven by weather phenomena (Patz et al. 2008) or accidental point-source input—e.g., sewer overflow, especially in the case of combined sewer systems (McLellan et al. 2007)—may also have a significant impact on recreational water quality in general, or at specific bathing sites. However, the monitoring of such events is highly difficult due to their ad hoc characteristics (Nevers et al. 2014). In the present study, hotspots and the main drivers of continuous and occasional fecal pollution were identified in order to provide support for management practices in the case of a large shallow Central European lake intensively used for recreational purposes throughout the year (Wolfram et al. 2014). Peer-reviewed studies on the monitoring of long-term (decadal) developments and the drivers of fecal pollution levels in lakes are scarce, e.g., Uejio et al. (2012), Weiskerger and Whitman (2018), and Whitman et al. (1999). It is to be suspected that the main reason for this lies in the lack of academic interest and the difficulties of financing scientific monitoring studies over and above routine surveillance programs or complex studies on pollution source identification. Such investigations are, however, a prerequisite for effective bathing water management and decision-making, e.g., beach closure (Olyphant 2003; Uejio et al. 2012; Whitman 2003; Whitman et al. 1999). For example, despite the fact that at the largest shallow freshwater lake in Central Europe, Lake Balaton (Istvánovics et al. 2007), there are > 140 official EU bathing sites with monitoring stations (EEA 2018), scientific publications on the assessment of SFIB variance are so far lacking. With regard to general water quality parameters (physical, chemical, and biological), the situation is somewhat better. The monitoring data is assessed to a higher extent, with a significantly larger number of publications available, but still, the amount of information gained from the analyses has not kept pace with the increasing amount of data (Kovács et al. 2012b).

Methodological approaches similar to those employed in the present study can be found; in these, too, the statistical relationship of fecal indicator bacteria and independent meteorological and water quality parameters are assessed to model SFIB variance (Nevers et al. 2014; Nevers and Whitman 2005) and derive a predictive model (Dada and Hamilton 2016; Jones et al. 2013; Uejio et al. 2012). However, these studies do not consider multiple sites together to obtain an overall picture of a lake, and except the long-term data on Geneva Lake, in Wisconsin, USA (Uejio et al. 2012), they cover a much smaller time interval than the present study.

### Hotspots of fecal pollution

Descriptive statistics have already revealed that the sampling sites near settlements, especially at Rust and the WWTP outflow at Podersdorf, are the most affected by fecal pollution. By way of contrast, the sites in the open water area of the lake are occasionally affected by fecal pollution, although their background levels are generally low. All the official EU bathing sites at the lake meet the requirements of the EU Bathing Water directive, standards which are less strict than in, e.g., the United States (US EPA 2012) or Canada (Health Canada 2012), due to differences in legislation. According to the US EPA recreational water criteria, the statistical threshold values (which approximate the 90th percentile of the water quality distribution) for *E. coli* and enterococci are 410 and 130 CFU 100 ml^−1^, respectively (US EPA 2012), in comparison to 900 and 330 CFU 100 ml^−1^ according to the European Bathing water directive (EC 2006).

With the exception of four sites, all other sampling sites (*n* = 15) complied with the guideline values for good water quality, and 13 sites even displayed excellent water quality (Fig. 1). No difference was observed between the two time periods considered (bathing season June–August versus March–October; Table S3). This indicates that the influence of the bathers during the bathing season is of negligible importance for the water quality of the lake and other influencing factors (see below) prevail.

By assessing the SFIB variability in the lake, hotspots of fecal pollution were identified coinciding with sites at which elevated anthropogenic activity is present (Magyar et al. 2013). The two sub-regions most influential in terms of the variance of EC and ENT in the Austrian part of the lake are located at Rust and Podersdorf (Fig. 2a, b), with multiple sites identified as hotspots. At Rust, only site 9 was not identified as a hotspot, but it should be noted that the loading of site 9 in the first principal component for EC (0.68) is just below the chosen threshold of 0.7. At Podersdorf, all of the sites were identified as EC hotspots except for site 32, located in a bay sheltered from the main currents (Józsa et al. 2008). Site 25, although located near Podersdorf, was not found to be a hotspot of fecal pollution since it is mainly influenced by the waters of the Golser Channel. Although the channel brings treated wastewater from Mönchhof and Gols, due to the retention ponds on the bank of the Neusiedler See (on the landside edge of the reed belt) and the long residence time through the densely vegetated channel, the impact of the treated wastewater on microbiological water quality is clearly dampened.

The other hotspots (sites 1, 20, 22, and 23; Fig. 2c), despite showing comparable SFIB concentrations to those in the reed belt and/or the open water (e.g., Table 1, Fig. 2c), still have a significant amount of SFIB variance (Fig. 2a,b), in most cases caused by the improper handling of wastewater (Belmont et al. 2004; Burian et al. 2000; Wang et al. 2014). Specifically, in the bay of the Biological Station (site 1) and the channel at Neusiedl (site 22), numerous sailing boats are present during the recreational season. In Jois (site 20) and Weiden (site 23), permanent residences with improper wastewater handling may be the source of the SFIB variance. In the case of Jois, these are located in a rather closed bay, while at Weiden these are found in the corridor of the channel in front of which the measurements are conducted (Fig. 2c).

### Meteorological and water quality factors driving microbial fecal pollution in Neusiedler See

Meteorological events are known to have a significant impact on the microbial pollution status of surface waters (Patz et al. 2008). Storm events, sewer overflow, or increased surface run-off due to heavy precipitation events lead to short-term increases in fecal indicator concentrations. Similar phenomena were expected in the case of the Neusiedler See, and all the more so, as the lake is very shallow and dilution of fecal pollution is less effective. In fact, some significant correlations were found between SFIB variance and meteorological parameters. On the one hand, the observed negative correlations between the SFIB variance and particular meteorological parameters (Table 2) imply that if there is no wind/precipitation, the water and air temperature are more likely to be higher, along with a greater chance of an elevated number of sunny hours. In these cases, peaks in SFIB presence are less likely to occur, as was also found to be so in the case of the Great Lakes (Nevers et al. 2014). On the other hand, SFIB correlated with wind speed in a positive way (Tables 2 and 3). For both EC and ENT, the Northwesterly wind direction was found to be the most determining (Fig. 3). This concurs with the wind directions (N, NNW, NW, SSE) observed to be dominant in the joint campaign of Józsa et al. (2008), consisting of on-site measurements. In the case of the shallow waters at the Podersdorf beach, wave action caused by the onshore winds results in the resuspension of the sediment. Recent studies along the coast of Lake Michigan indicate that the bacteria concentration has grown so high in the sand of the beaches that wave action can stir up the sediment and temporarily release bacteria into the water. This, in turn, resulted in high concentrations of *E. coli* (Olyphant 2003; Whitman 2003). Such scenario may also happen along the Podersdorf beach as well, though unfortunately no recent data are available on the bacteria concentrations in the sediments (Dokulil 1975).

In the case of heavy rains, mostly accompanied by NW winds, the WWTP effluent channel at Podersdorf overflows, accounting for SFIB peaks at the bathing sites. Urban stormwater has emerged as a major contributor of bacterial and chemical pollution in watersheds (McLellan and Jensen 2003), especially in the case of combined sewer systems, where heavy rainfall exceeds the holding capacity of the system and a mixture of sanitary sewage and stormwater is discharged into the lake. In Podersdorf, for example, 97% are part of a combined sewer system (Wolfram et al. 2014).

In the case of the second most important hotspot, Rust, it had already been shown previously that in terms of general water quality data, on account of the increased recreational usage and possibly improper handling of domestic sewage water in the area, private weekend huts cause pollution patterns different from, e.g., the open water or the reed belt (Magyar et al. 2013). Although the obtained principal components in the present study represent the whole lake, the SFIB variance at Rust was highly determining (Fig. 2a, b). It is assumed that this is directly related to the weekend huts, as also in the case of the general water quality parameters (Magyar et al. 2013). Thus, if it rains, anthropogenic presence/activity decreases, lowering the amount of fecal input at Rust, manifested in the significant negative relationship between precipitation at Rust and the EC and ENT variance (Table 2).

The significant positive relationship between nitrate concentrations and SFIB variance may be due to diffuse pollution from livestock and horses (Canter 1996) in the area. No significant correlations were found between the discharge of the main tributary, the Wulka, and of the water level, with the fecal pollution at the hotspots. Despite the fact that the Wulka transports the wastewater from several WWTPs along the river, its influence on microbial lake water quality on an annual scale is negligible. This can be explained by the fact that the water of the Wulka passes through a 3-km-wide reed stand before reaching the open water of the lake (Dinka et al. 2016). Also, annual water level fluctuations seem to be unimportant despite the fact that a concentration of fecal pollution at lower water levels may be assumed. In contrast, water level changes may be relevant at the short time scale when strong winds induce increased wave action leading to resuspension of sediments and—in case of westerly winds at the Wulka Delta—mobilization of fecal bacteria retained in the red belt into the lake.

Differences in the correlation patterns (more EC than ENT hotspots were found) can be explained by the greater persistence of enterococci in comparison to *E. coli*, especially in sub-saline environments (Jin et al. 2004; Kirschner et al. 2014). Thus, EC variability (input and die-off) is more determined by meteorological and local effects (~ 42% explained by the first PC) than ENT (~ 33%). Consequently, further investigations are needed to determine the main controlling factors of ENT variability at the sites which are solely EC hotspots.

### Geostatistical assessment of SFIB variance

Overall, it can be concluded that with an approximately 1 km spatial range, interpolation was insufficient in the case of distant sites with the current sampling grid, and that in general the sites are mostly representative of local phenomena. Using a rigorous method to determine homogeneous groups of sampling sites (Kovács et al. 2014), concurring results were obtained with the present ones. In a particular study based on multiple water quality variables, most of the sampling sites of the lake formed separate groups. However, in the areas of the hotspots determined and discussed in the present paper, homogeneous groups of multiple sampling sites were observed (Kovács et al. 2014). Thus, the vicinity of the main hotspots (e.g., Podersdorf and Rust)—where multiple sites were located within the spatial range—was represented by “evaluable” isolines, not only bull’s eyes (Fig. 4). In addition, a similar pattern was observed on the isoline map of the scores of the first principal component of general water quality variables (mainly inorganic ones), clearly indicating the anthropogenic influence on the water quality of the lake at Rust and by the Podersdorf WWTP (Magyar et al. 2013).

The extension of the influence of the fecal pollution hotspots (Fig. 4) is comparable to those modeled in the surface sediments of Bay of Vidy on Lake Geneva (Switzerland), where the WWTP’s outlet was close to the shore in a shallow area (Poté et al. 2008). From a methodological aspect, we presume that the maps from Lake Geneva were also modeled using variography and kriging. However, due to the lack of a documented semivariogram model, the actual representativity and the spatial correlation structure of their data (Poté et al. 2008) cannot be evaluated, unlike in the present case. In cases where the subject of the spatial modeling was bacteriophage tracer tests—considered to be consistent with the spatiotemporal variability of SFIB (Goldscheider et al. 2007)—interpolation with kriging was not possible due to the lack of homogeneous mixing processes (Goldscheider et al. 2007; Wanninkhof et al. 2005). At the Neusiedler See hotspots, the number of investigated sampling locations was lower than in the previously mentioned studies. Nevertheless, interpolation was sufficient, probably due to the steady-state flow patterns in the lake (*v* = 10 m s^−1^) in the area (Józsa et al. 2008). This suggests that from a scientific point of view, a denser and planned campaign—using a quasi-equidistant grid—should be commenced to map the SFIB variance over the whole lake, for example, a couple of times prior to and then after storm events in the course of one bathing season. In this way, the spatial SFIB variability over the whole lake, but especially at the hotspots, could be sufficiently determined. Then, from a practical point of view, the obtained results could be used to plan and set up an economically optimized/recalibrated monitoring system, which would reliably represent SFIB variance over the whole lake, at the same time allowing for a special focus on the problematic areas with alarming SFIB variance.

## Conclusions

For lakes intensively used for recreation, specific microbiological water quality targets have to be met. Thus, spatiotemporally representative surveillance needs to be conducted spanning a set of various biotic and abiotic water quality parameters. However, such surveillance may be in itself insufficient to elucidate the main drivers of fecal pollution events in a complex lake/watershed ecosystem. The in-depth analysis of the microbial fecal pollution data at hand coupled with additional environmental variables determined at a large number of sampling sites is needed. In the present study, with the application of multivariate statistical and geostatistical methods, the hotspots of standard fecal indicator bacteria (SFIB) variability were identified and delineated in space in a highly important recreational shallow lake in Europe. The presence of the hotspots was demonstrated to be attributable to the presence of extensive anthropogenic activity, such as the emission of effluents from wastewater treatment plants or the improper handling of communal sewage. At sites located remotely from the fecal pollution hotspots, while continuously low SFIB levels were to be observed, peaks of SFIB presence were noted, underlining the affected state of the whole lake. Thus, a greater effort is required to reduce fecal pollution inputs at the hotspot sites.

Based on the geostatistical analysis, it is suggested that an intense campaign be initiated, both in time and space, in order to be able to map the different areas/habitats of the lake more representatively in terms of SFIB. Were any such campaign to be planned, the prevailing wind directions (mostly northerly) should be taken into account. With the use of variography, the present grid explicitly indicates the areas as yet uncovered by the current network of sampling sites. On the basis of this information, a sustainable, economically optimized monitoring program could be set up, surpassing the current surveillance activities at the seven Austrian EU bathing sites with the aim of improving recreational water quality over the whole lake.

In addition to information on the extent and spatiotemporal patterns of recent fecal pollution based on *E. coli* and intestinal enterococci concentrations, more persistent fecal pollution indicators and microbial source tracking techniques—e.g., Edge et al. (2010), Kirschner et al. (2017), and Reischer et al. (2011)—should be applied to elucidate the patterns and identify the main drivers of and contributors to fecal pollution in complex lake/watershed ecosystems. In the area under consideration, the implementation of *Clostridium perfringens* or coliphages into the monitoring concept and the application of human-, horse-, pig-, bird-, and ruminant-associated microbial fecal pollution markers for source tracking purposes would significantly improve the understanding of the observed complex pollution patterns and efficiently support bathing-water management practices.

## Electronic supplementary material

ESM 1 (DOCX 3.90 mb)
