# Supplementary material for: Hotspots and main drivers of fecal pollution in Neusiedler See, a large shallow lake in Central Europe
Source: Environ Sci Pollut Res Int. 2018 Aug 13;25(29):28884–98. doi: 10.1007/s11356-018-2783-7 (PMC6153677; doi:10.1007/s11356-018-2783-7)
Supplement: Supplementary file 1 — (DOCX 3.90 mb) [file 11356_2018_2783_MOESM1_ESM.docx]

**Supplementary Online Material**

**Hatvani IG, Kirschner AKT, Farnleitner AH_,_ Tanos P & Herzig A**

Hotspots and main drivers of fecal pollution in Neusiedler See, a large shallow lake in Central Europe

**
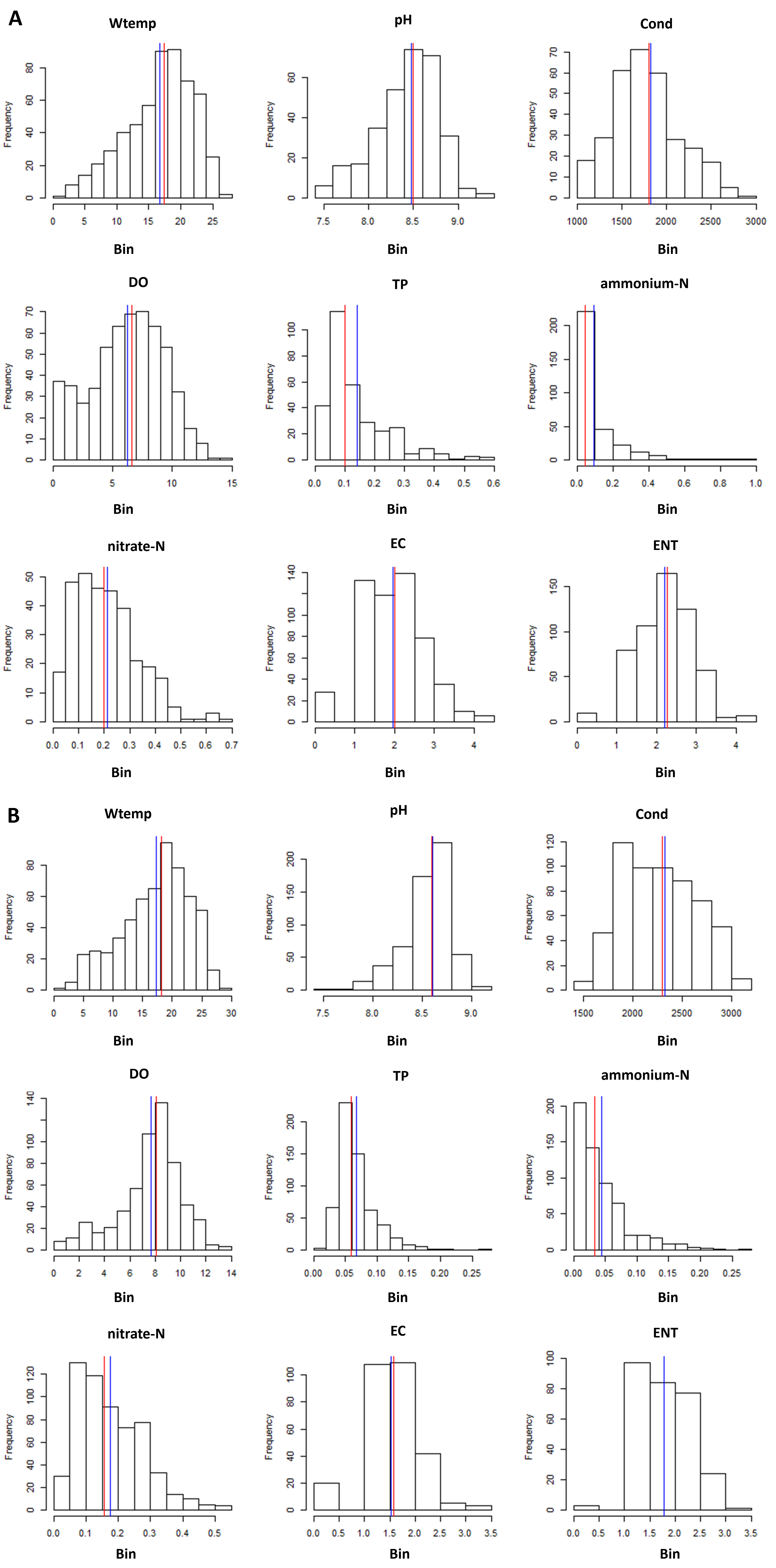
**

**Fig. S1. Histograms of the WQP and SFIBs at site 29 (WWTP outflow) A) and an open water site (site 33) B). The mean and median values are indicated with the blue and red vertical lines respectively.**

**Table S1**: List of sampling sites selected for statistical analysis

| Site - nr | name | description |
| --- | --- | --- |
| 1 | Biological Research Station | In the middle of the bay by the Biological Station |
| 2 | Illmitz | EU-bathing site |
| 5 | Open lake South | Open lake in the southern part of the lake |
| 6 | Mörbisch | EU-bathing site |
| 7 | Rust South | Site near to bathing huts |
| 8 | Rust | EU-bathing site |
| 9 | Rust North | Site near to bathing huts |
| 11 | Reed belt West | Site next to reed belt, West |
| 13 | Wulka | Site next to inflow of the River Wulka |
| 16 | Purbach channel | Shipping channel through the reed belt |
| 17 | Purbach | Site next to channel outlet |
| 18 | Breitenbrunn | EU-bathing site |
| 19 | Breitenbrunn-harbour | Site next to sailing yacht harbor |
| 20 | Jois | Sailing yacht harbor |
| 21 | Neusiedl-harbour | Sailing yacht harbor |
| 22 | Neusiedl | EU-bathing site; at a channel coming from Neusiedl |
| 23 | Weiden | EU-bathing site |
| 24 | Open lake North | Open lake in the northern part of the lake |
| 25 | Gols channel | Influent of the Gols wastewater channel |
| 26 | Podersdorf | EU-bathing site |
| 27 | Open lake middle | Open lake, near the middle |
| 28 | Reed belt East | Site next to reed belt, East |
| 29 | Podersdorf-WWTP1 | Main inflow of WWTP effluent at Podersdorf |
| 30 | Podersdorf-WWTP2 | Inflow of WWTP effluent at Podersdorf |
| 32 | Podersdorf North | Bathing site at Podersdorf North |
| 33 | Rust channel | Site next to former wastewater channel at Rust |

**Conversion of *E. coli* concentrations determined according to ISO 7899-2:2000 before 2003 to *E. coli* values determined by Colilert (after 2003)**

Since there was a change in the measurement method for EC in 2003, it became necessary to convert the *E. coli* concentrations determined according to ISO 7899-2:2000 before 2003 to *E. coli* values as determined by Colilert (ISO 9308-2:2012), based on results from parallel measurements conducted in 2003.

**Defining the regression model**

As a first step a segmented regression was applied using SegReg Software to the logarithmic data of the available parallel measurements between the ISO 7899-2:2000 and the Colilert method, with the latter as the independent variable. It indicated that there is a breakpoint (Bp) in the data x= 1.85 i.e. (10^1.85^-1) = 67.79 CFU/100 ml based on 125 pairs of data (**Fig. S2a**). For verification a segmented regression was prepared on the data above the breakpoint (above 70 CFU/100 ml; **Fig. S2b**). There is indeed no breakpoint above 70 CFU/100 ml.


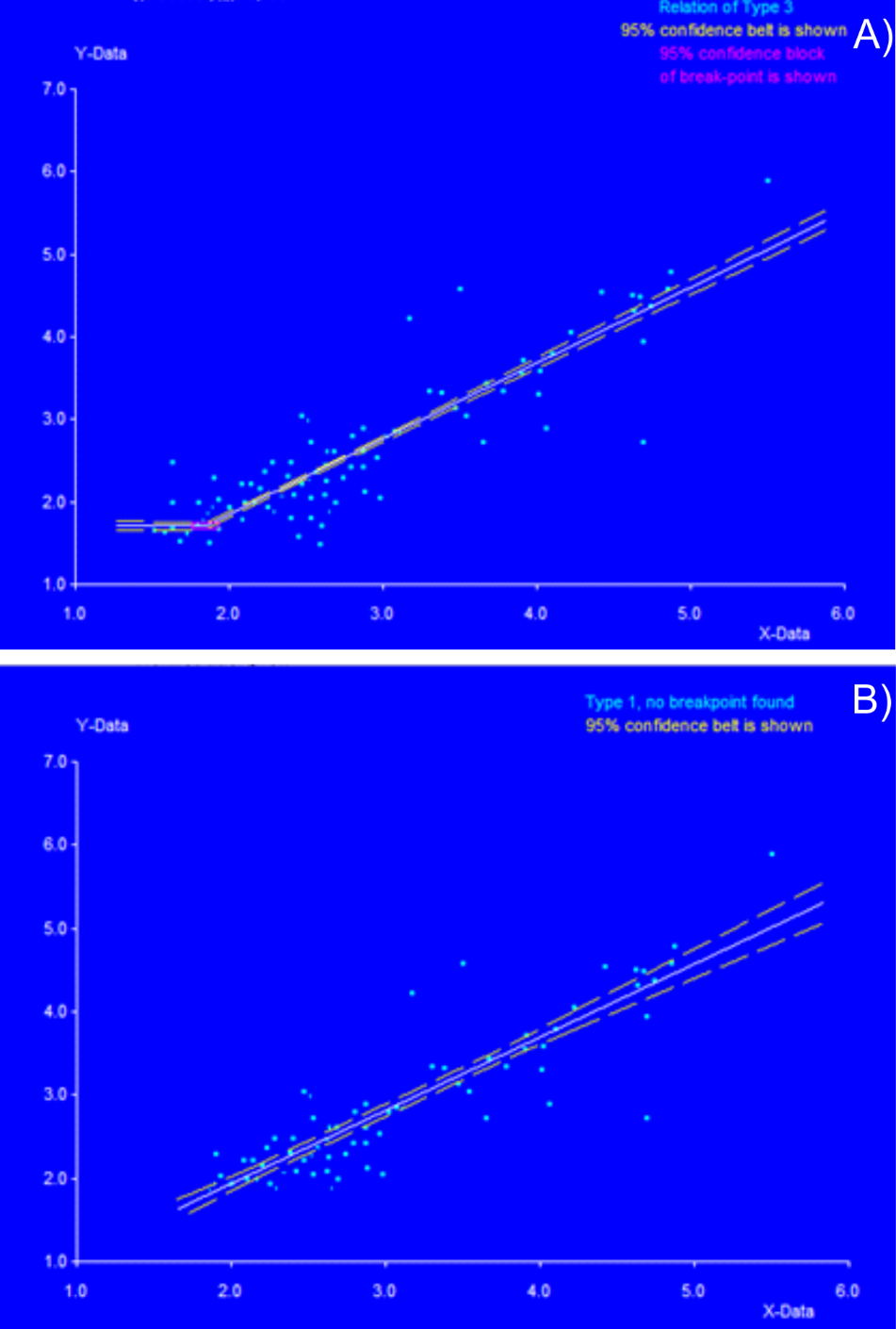


**Fig. S2**. Segmented regression of (A) the parallel measurements excluding the values under the limit of quantification (15 CFU/100 ml) showing the breakpoint at x=1.85, and (B) of the parallel measurements on the data above the breakpoint of 70 CFU/100 ml = x=1.85. All data log_10_ transformed. X-axis: ISO 7899-2:2000 data; Y-axis: Colilert data

As a second step a robust regression was prepared on the data above the breakpoint to obtain the regression model to convert the ISO 7899-2:2000 data to the Colilert method (**Eqn. S2** & **Fig. S3**)

*y=0.8962064*x+0.1294463* **(S1)**

*r^2^=0.7956 (significant at α=0.0001)*


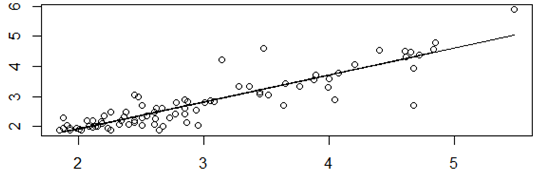


*E. coli* after Colilert

[log (MPN/100ml)]

*E. coli* after ISO 7899-2:2000 [log (CFU/100ml)]

**Fig. S3** Robust regression on the parallel data above 70 bacteria between ISO 7899-2:2000 data and the Colilert data as dependent variable

**Table S2.** Sites (marked with a cross) with the meteorological parameters examined between 1992 and 2013. NDL: Neusiedl; PD. Podersdorf; Aptl: Apetlon; Wdn: Weiden; Biol. Stat: Biological Station Illmitz.

| **Parameters** | | **Meteorological stations** | | | | | |
| --- | --- | --- | --- | --- | --- | --- | --- |
|  |  | **NDL** | **Rust** | **PD** | **Aptl** | **Wdn** | **Biol.Stat** |
| Air temperature | T_air_ [°C] | + |  |  |  |  | + |
| Precipitation (daily sum) | Prec [mm day^-1^] | + | + | + | + | + |  |
| Sunny hours | Sh [h] | + |  |  |  |  |  |
| Global radiation | Rad [J cm^-2^] | + |  |  |  |  |  |
| Wind speed (daily average) | VV [m s^-1^] | + |  |  |  |  |  |
| Max wind speed | VV_max_ [m s^-1^] | + |  |  |  |  | + |

**Table S3.** 90- and 95 percentiles from the single observations of the EC and ENT from Lake Neusiedlersee for the whole investigated time interval (1 Mar – 31 Oct) and the bathing season only (1 Jun – 31 Aug 1992-2013). The guideline values (in log): EC: exc - 2.7 (q95), good - 3.0 (q95), sufficient - 2.95 (q90); ENT: exc - 2.3 (q95), good - 2.6 (q95), sufficient - 2.51 (q90) (EC, 2006). Asterisks (*) mark the EU bathing sites.

**Wind direction conversion**

During the data preparation, the wind direction data obtained from the Austrian Zentralanstalt für Meteorologie und Geodynamik had to be preprocessed and converted. The numerical wind directions were converted into string variables, since the original numeric records split a circle into 32 sectors starting over at N (360°/32=11.25°). Thus, each direction (4 sectors) was given a string codename, e.g. 2-6:NE; 6-10:E or 30-2:N (**Fig. S4**)


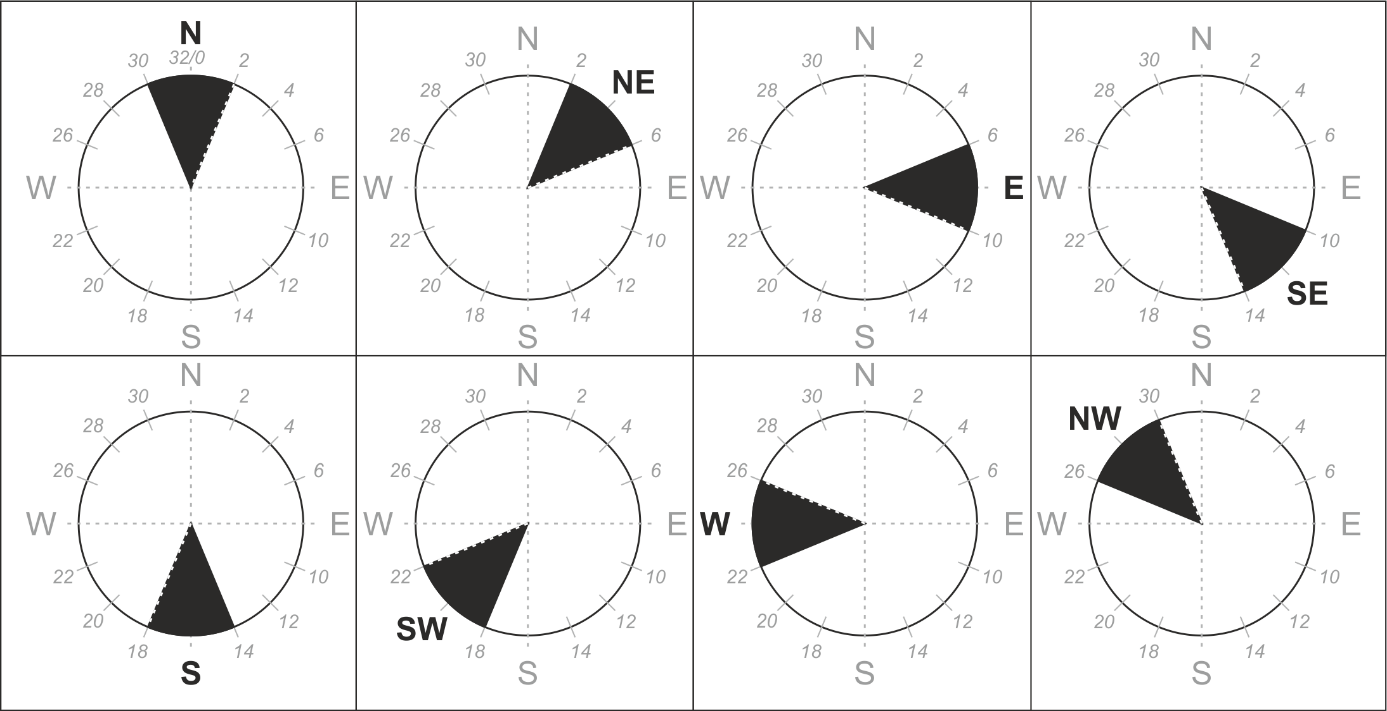


**Fig. S4.** Conversion of numerical wind direction into text, for easier use and better interpretation. The broken line represents the upper boundary /x<value/

Wind directions were measured three times a day. So, if at least two out of the three daily wind direction measurements were related to e.g. N, such as NW, N or NE, then the corresponding wind speed was taken as if it was N* (**Fig. S5**: leftmost panel) and is called directional wind speeds in the study. Consequently, the directional wind speeds were averaged annually for further calculations.


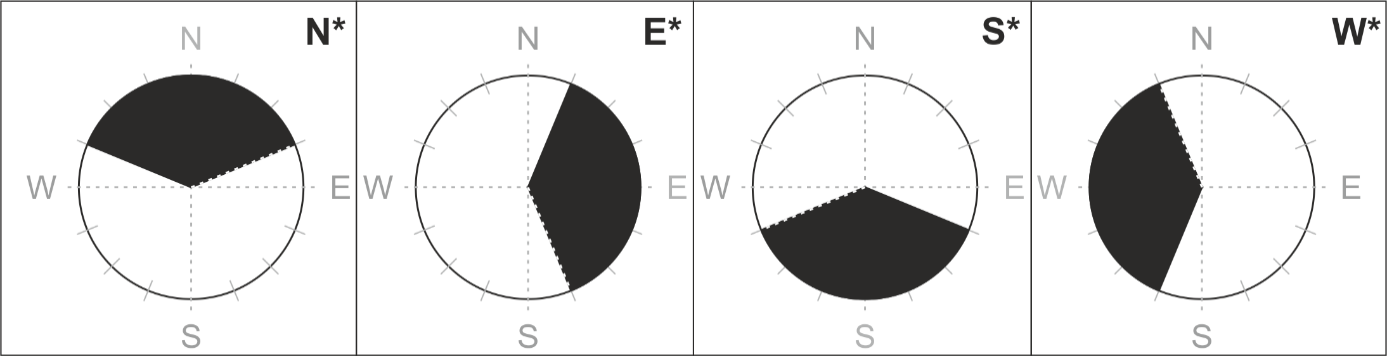


**Fig. S5.** Describing the simplified approach to deal with the wind directions to be able to use them not only in an empirical, but in a stochastic way, applying statistics. The asterisk (*) mark the simplified version of the wind directions.

Note here, that in this analysis, placing the wind speeds on a finer scale (like in **Fig. 2**) i.e. using exact matches, was a dead end. If the constraint was that two out of the three cases of the recorded wind direction had to be exactly N or exactly NW, the number of measurements available for the analysis was extremely low compared to the total dataset (e.g. N: 10%; NE: 3%; E:3%, etc.; average for all the “exact matches”: 9%, while the total was: 5391 data). On the contrary, in the case of the applied approach (Fig. 7) these were: N*:55%; E*:25%; S*:28%; W*:46%.


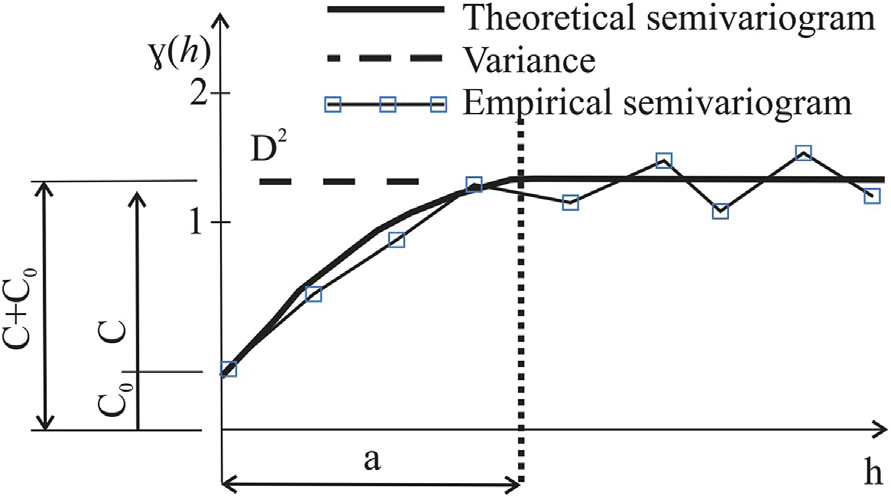


Fig. S6. Properties of the semivariogram, where “a” stands for range, “C” for the reduced sill and “C_0_” for the nugget effect, “h” for lag distance and “D^2^” for the variance (taken from Hatvani et al. 2017)

**References**

EC (2006): DIRECTIVE 2006/7/EC OF THE EUROPEAN PARLIAMENT AND OF THE COUNCIL of 15 February 2006 concerning the management of bathing water quality and repealing Directive 76/160/EEC. Official Journal of the European Communities L 64, 1-37

Hatvani IG, Leuenberger M, Kohán B, Kern Z (2017) Geostatistical analysis and isoscape of ice core derived water stable isotope records in an Antarctic macro region Polar Science 13:23-32 doi:https://doi.org/10.1016/j.polar.2017.04.001
